# Early-onset tufting enteropathy in HAI-2-deficient mice is independent of matriptase-mediated cleavage of EpCAM

**DOI:** 10.1242/dev.201801

**Published:** 2023-08-31

**Authors:** Roman Szabo, Makiko Kawaguchi, Hiroaki Kataoka, Thomas H. Bugge

**Affiliations:** ^1^ Proteases and Tissue Remodeling Section, National Institute of Dental and Craniofacial Research, National Institutes of Health, Bethesda, MD 20892, USA; ^2^ Section of Oncopathology and Regenerative Biology, Department of Pathology, Faculty of Medicine, University of Miyazaki, Miyazaki 8891692, Japan

**Keywords:** Enteropathy, Epithelial barrier, EpCAM, HAI-2, Intestinal development, Membrane-anchored serine protease

## Abstract

Congenital tufting enteropathy (CTE) is a life-threatening intestinal disorder resulting from loss-of-function mutations in *EPCAM* and *SPINT2*. Mice deficient in *Spint2*, encoding the protease inhibitor HAI-2, develop CTE-like intestinal failure associated with a progressive loss of the EpCAM protein, which is caused by unchecked activity of the serine protease matriptase (ST14). Here, we show that loss of HAI-2 leads to increased proteolytic processing of EpCAM. Elimination of the reported matriptase cleavage site strongly suppressed proteolytic processing of EpCAM *in vitro* and *in vivo*. Unexpectedly, expression of cleavage-resistant EpCAM failed to prevent intestinal failure and postnatal lethality in *Spint2*-deficient mice. In addition, genetic inactivation of intestinal matriptase (*St14*) counteracted the effect of *Spint2* deficiency in mice expressing cleavage-resistant EpCAM, indicating that matriptase does not drive intestinal dysfunction by excessive proteolysis of EpCAM. Interestingly, mice expressing cleavage-resistant EpCAM developed late-onset intestinal defects and exhibited a shortened lifespan even in the presence of HAI-2, suggesting that EpCAM cleavage is indispensable for EpCAM function. Our findings provide new insights into the role of EpCAM and the etiology of the enteropathies driven by *Spint2* deficiency.

## Teaser

**Highlighted Article:** Cleavage-resistant EpCAM does not prevent intestinal failure in the *Spint2*-deficient mouse model of congenital tufting enteropathy, challenging the current model of proteolysis-driven disease progression.

## INTRODUCTION

The intestinal epithelium represents a crucial barrier that protects the body from potentially harmful foreign substances and waste products as well as a complex microflora of commensal and pathogenic bacteria found in the lumen of the gastrointestinal tract. When compromised, an increase in paracellular flux across the epithelium can have negative effects on the processing and absorption of nutrients, electrolytes and water, resulting in malnutrition. In addition, a leaky barrier often triggers an uncontrolled immune reaction, resulting in intestinal inflammation and possibly extraintestinal autoimmune and metabolic disorders, such as rheumatoid arthritis or diabetes (Chelakkot et al., 2018; Groschwitz and Hogan, 2009).

Congenital tufting enteropathy (CTE; OMIM #613217) is an early-onset severe intestinal insufficiency characterized by epithelial dysplasia, villous atrophy and a compromised intestinal epithelial barrier, leading to chronic watery diarrhea, dehydration and failure to thrive in the absence of parenteral feeding (Sivagnanam et al., 2008). A systematic study of individuals diagnosed with CTE revealed that more than 70% of cases are associated with bi-allelic mutations in the *EPCAM* gene, encoding the epithelial cell adhesion molecule (EpCAM) (Sivagnanam et al., 2008; Salomon et al., 2014; AlMahamed and Hammo, 2017; Pathak et al., 2019). EpCAM has been reported to regulate epithelial cell physiology by maintaining the cell-surface pool of the tight junction protein claudin-7 (CLDN7), and possibly other claudins, thus promoting epithelial cell-cell adhesion and barrier formation (Wu et al., 2017; Chen et al., 2021; Higashi et al., 2023; Szabo et al., 2022). Consequently, loss of intestinal barrier function because of destabilization of tight junctions is considered to be the chief contributor to the diarrheal disease in individuals deficient for EpCAM. The majority of individuals with CTE that do not carry mutations in the *EPCAM* gene present with a syndromic form of CTE (sCTE), also termed congenital sodium diarrhea (OMIM #270420), which is caused by loss-of-function mutations in the *SPINT2* gene (Salomon et al., 2014; Heinz-Erian et al., 2009; Holt-Danborg et al., 2019). Unlike conventional CTE, in which the pathology appears to be confined to the epithelia of small and large intestines, sCTE is typically associated with a variety of extraintestinal phenotypes, most typically choanal or anal atresia, keratitis, hypertelorism and pillar dysplasia (Salomon et al., 2014).

*SPINT2* encodes the transmembrane Kunitz-type serine protease inhibitor HAI-2, which is widely expressed across human and mouse epithelia (Szabo et al., 2008; Itoh et al., 1999). HAI-2 is crucial for mouse embryonic development, owing to its role in the regulation of proteolytic activity of the trypsin-like serine proteases matriptase (ST14) and prostasin (PRSS8) (Szabo et al., 2009). Embryonic lethality in HAI-2-deficient mice can be circumvented by intestine-specific genetic ablation of *Spint2* or by restricting the activity of matriptase/prostasin pathway. However, loss of HAI-2 in these mice leads to a CTE-like intestinal insufficiency characterized by a widespread villous atrophy, enterocyte tufts, epithelial erosion and bleeding into the intestinal lumen (Kawaguchi et al., 2019; Szabo and Bugge, 2018).

Because of the close similarities between the clinical, histological and molecular presentation of CTE and sCTE, it was recently proposed that an increase in EpCAM cleavage and degradation, owing to excessive protease activity resulting from the absence of HAI-2, is responsible for the CTE symptoms in HAI-2-deficient individuals and mice (Wu et al., 2017; Kawaguchi et al., 2019; Szabo and Bugge, 2018). Indeed, matriptase efficiently cleaved EpCAM within the extracellular thyroglobulin-like (TY) domain at Arg80 when co-expressed in kidney and intestinal epithelial cells, and this cleavage led to rapid internalization and lysosomal degradation of EpCAM and claudin-7 (Wu et al., 2017). In support of the proposed model, inactivation of matriptase prevented loss of epithelial integrity caused by HAI-2 deficiency in intestinal organoid culture and dramatically delayed onset and severity of intestinal defects in HAI-2-deficient mice (Szabo et al., 2019; Kawaguchi et al., 2019).

In this study, we report that the loss of HAI-2 indeed leads to an increased, matriptase-dependent proteolytic processing of EpCAM. However, using newly generated mouse strains expressing cleavage-resistant EpCAM protein variants that lack the predicted matriptase cleavage site, we show that the suppression of EpCAM cleavage fails to rescue or suppress any of the intestinal phenotypes observed in HAI-2-deficient mice. We also provide evidence that the cleavage of EpCAM may, in fact, be crucial for its physiological function.

## RESULTS

### Loss of HAI-2 leads to increased matriptase-mediated cleavage of EpCAM *in vivo*

Increased cleavage and the resulting loss of EpCAM function were proposed as the mechanism leading to intestinal insufficiency in CTE individuals carrying *SPINT2* mutations (Wu et al., 2017; Das et al., 2021). Indeed, intestinal defects in two recently developed *Spint2*-deficient mouse models of CTE are associated with a substantial decrease in detectable EpCAM protein and a loss of its membrane localization (Szabo and Bugge, 2018; Kawaguchi et al., 2019). Furthermore, expression of the HAI-2 target protease, matriptase, was shown to trigger rapid cleavage and internalization of EpCAM in cultured epithelial cells (Wu et al., 2017, 2020).

**Fig. 1.**
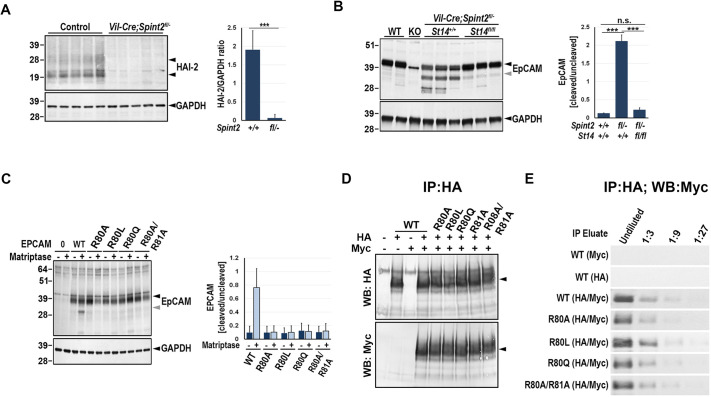
**Arg 80 is crucial for matriptase-mediated processing of EpCAM.** (A) Western blot analysis of the expression of HAI-2 protein in the intestinal tissues from newborn wildtype (control, *n*=5) and HAI-2 conditional knockout (*Vil-Cre^+/0^;Spint2^fl/−^*, *n*=5) mice. No HAI-2 protein was detected in tissues from *Vil-Cre^+/0^;Spint2^fl/−^* mice. (B) Representative western blot (left) and quantification (right) of the ratio of the signal corresponding to cleaved versus uncleaved EpCAM in intestines from 5-day-old wildtype (WT, *n*=4), *Epcam^−/−^* (knockout or KO, *n*=2), HAI-2 single-deficient (*Vil-Cre^+/0^;Spint2^fl/−^;St14^+/+^*, *n*=6), and HAI-2 and matriptase double-deficient (*Vil-Cre^+/0^;Spint2^fl/−^;St14^fl/fl^*, *n*=6) mice. Results show increased cleavage of EpCAM in HAI-2-deficient intestines, which is suppressed by inactivation of matriptase. (C) Representative western blot (left) and corresponding quantification (right, three independent experiments) of EpCAM protein in HEK293T cells expressing wildtype (WT), R80A, R80L, R80Q and R80A/R81A variants of human EpCAM alone or in combination with human matriptase. Mutation of Arg80 prevented cleavage of EpCAM by matriptase. (D) Analysis of dimerization of EpCAM variants by co-immunoprecipitation (IP). HEK293T cells were co-transfected with HA- and/or Myc-tagged wildtype WT, R80A, R80L, R80Q and R80A/R81A EpCAM variants. The cell lysates were immunoprecipitated with anti-HA antibodies, followed by a detection of HA (top) or Myc (bottom) epitopes using western blotting (WB). Co-immunoprecipitation of HA- and Myc-tagged proteins was detected for all EpCAM variants, indicating their ability to dimerize. (E) Western blot of Myc-tagged EpCAM protein in 1:3 serial dilutions of anti-HA IP eluate shown in D. No clear difference in the amount of Myc-tagged EpCAM pulled down by an anti-HA antibody was detected for any of the EpCAM variants. In A-D, positions of the two forms of HAI-2 (A), full-length EpCAM (B-D) and GAPDH (A-D) proteins are indicated with black arrowheads. Positions of the proteolytically-processed EpCAM (B,C) are indicated with grey arrowheads. Molecular mass markers (in kDa) are indicated on the left. n.s., not significant; ***P<0.001.

To ascertain whether the loss of HAI-2 is associated with an increased cleavage of EpCAM protein *in vivo*, we generated *Vil-Cre^+/0^;Spint2^fl/−^* mice with an intestinal epithelium-specific ablation of the *Spint2* gene. The inactivation resulted in an absence of detectable HAI-2 protein in the tissues of the small and large intestines (Fig. 1A). Western blot analysis of intestinal tissues from 5-day-old mice confirmed that the loss of HAI-2 did lead to a substantial increase in the proteolytic processing of EpCAM, as evidenced by a more than 15-fold increase in the ratio of cleaved to uncleaved EpCAM protein, when compared with that of wild-type littermate controls (Fig. 1B). Interestingly, the excessive cleavage of EpCAM was nearly completely suppressed in mice with genetic inactivation of intestinal matriptase (*Vil-Cre^+/0^;Spint2^fl/−^;St14^fl/fl^*), indicating that the protease is indeed largely responsible for the increased EpCAM cleavage in HAI-2-deficient intestines (Fig. 1B). These data demonstrate that the absence of HAI-2 is associated with a substantial increase in matriptase-mediated proteolytic processing of intestinal EpCAM.

### Arg80 is crucial for matriptase-mediated cleavage *in vitro* and the physiological function of EpCAM *in vivo*

In their previous work, Wu and colleagues used N-terminal sequencing to identify Arg80 within the extracellular TY domain of the EpCAM protein as the principal cleavage site used by trypsin-like serine proteases, such as matriptase (Wu et al., 2017). To directly test the role of Arg80 in the proteolytical processing of EpCAM, we transfected HEK293T kidney epithelial cells with expression plasmids encoding variants of human EpCAM in which the crucial Arg80 was replaced with alanine (R80A), or with the sterically more comparable leucine (R80L) or glutamine (R80Q). As most trypsin-like serine proteases have strong preference for arginine at their cleavage sites, a variant in which both Arg80 and Arg81 were replaced with alanine (R80A/R81A) was included to eliminate the possibility of potential cleavage at the adjacent Arg81. Consistent with previously published data, matriptase activity in cells expressing wild-type EpCAM resulted in a cleavage product readily detectable by western blotting (Fig. 1C). In contrast, no cleavage was detected in cells that expressed the EpCAM variants (Fig. 1C). These data confirm Arg80 as the primary matriptase cleavage site *in vitro*.

Several studies have proposed that EpCAM has the ability to dimerize and that the *cis*-homodimer is in fact the biologically functional unit at the cell surface (Gaber et al., 2018; Pavšič et al., 2014). The dimerization is mediated in large part by a TY loop within the extracellular domain of EpCAM, which contains both Arg80 and Arg81 (Pavšič et al., 2014). To ensure that replacing these arginine residues does not negatively impact EpCAM's ability to dimerize, HEK293T cells were co-transfected with plasmids expressing C-terminally HA- or Myc-tagged EpCAM variants. The resulting cell lysates were then subjected to immunoprecipitation using an anti-HA antibody followed by western blot detection of Myc tags. For all the variants, HA-tagged monomers were able to readily pull down Myc-tagged monomers with efficiency comparable with that of the wildtype EpCAM (Fig. 1D,E). This indicates that the replacement of Arg80 and Arg81 did not have an appreciable impact on the ability of the EpCAM protein to form homodimers.

**Fig. 2.**
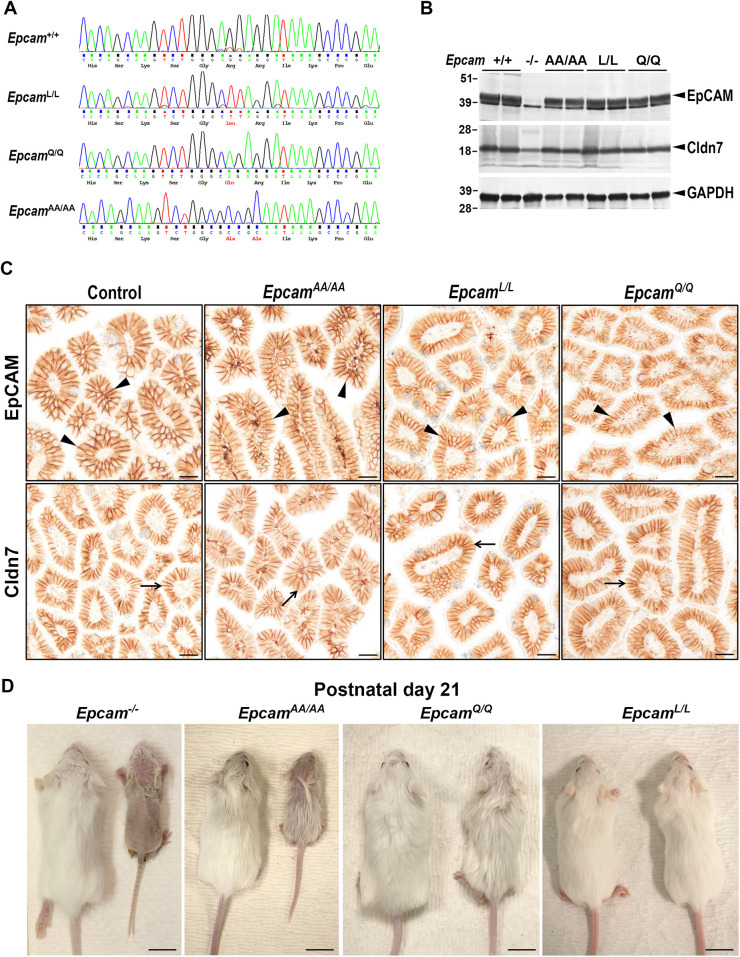
**Cleavage-resistant endogenous EpCAM variants show normal expression and cellular localization but compromised function *in vivo*.** (A) Representative chromatogram of DNA sequencing of mice expressing wildtype (*Epcam^+/+^*), R80L (*Epcam^L/L^*), R80Q (*Epcam^Q/Q^*) and R80A/R81A (*Epcam^AA/AA^*) variants of EpCAM protein confirming successful gene editing. Changes in amino acid sequences are indicated in red. (B) Representative western blot (from two independent experiments) of EpCAM (top), claudin-7 (Cldn7, middle) and GAPDH (bottom) in the intestines from 20-day-old mice expressing wildtype (+/+), R80A/R81A (AA/AA), R80L (L/L) or R80Q (Q/Q) variants of EpCAM. Intestines from *Epcam* null (−/−) mice were included as a control for antibody specificity. The expected positions of the full-length EpCAM, claudin-7, and GAPDH proteins are indicated with black arrowheads. Positions of molecular mass markers (in kDa) are indicated on the left. (C) Representative images (from at least five mice per genotype) of anti-EpCAM (top, arrowheads) and anti-claudin-7 (Cldn7, bottom, arrows) immunostaining of small intestines from 20-day-old mice showing basolateral membrane localization of wildtype (control), R80A/R81A (*Epcam^AA/AA^*), R80L (*Epcam^L/L^*) and R80Q (*Epcam^Q/Q^*) variants of EpCAM. Elimination of Arg80 did not lead to any substantial changes in the level of expression or cellular localization of EpCAM protein. Scale bars: 25 µm. (D) Overall outward appearance of 21-day-old littermate mice expressing wildtype (left) or mutated (right) R80A/R81A (*Epcam^AA/AA^*), R80L (*Epcam^L/L^*) and R80Q (*Epcam^Q/Q^*) EpCAM variants. Mice expressing R80A/R81A EpCAM present with alopecia and severe growth retardation comparable with *Epcam* null (*Epcam^−/−^*) animals, whereas *Epcam^Q/Q^* mice have a slightly smaller size and ruffled coat and *Epcam^L/L^* mice are comparable in appearance with their wildtype littermate controls. Scale bars: 1 cm.

We next used CRISPR/Cas9-based genome editing to generate three mouse knockin strains expressing the R80L, R80Q and R80A/R81A EpCAM variants (*Epcam^L/+^*, *Epcam^Q/+^* and *Epcam^AA/+^*, respectively), and confirmed successful modification of the nucleotide sequence in all three strains by DNA sequencing (Fig. 2A). *Epcam^L/L^*, *Epcam^Q/Q^* and *Epcam^AA/AA^* mice produced by heterozygous breeding all looked unremarkable at birth compared with wildtype littermate controls, and they displayed normal levels of membrane-associated EpCAM protein (Fig. 2B,C). Similarly, the intestinal expression and membrane localization of claudin-7 was unaffected, indicating that the elimination of Arg80 did not diminish the ability of EpCAM to stabilize claudin-7 on the cell surface (Fig. 2B,C). Histological analysis of intestinal tissues from 5-day-old mice did not reveal any obvious structural abnormalities in the small or large intestines of the *Epcam^L/L^* and *Epcam^Q/Q^* mice (Fig. S1). However, already at this age, *Epcam^AA/AA^* mice exhibited signs of villous atrophy in the small intestines and a reduction in the number of mucin-producing goblet cells in colon, similar to those in *Epcam* null mice (Fig. S1).

**Fig. 3.**
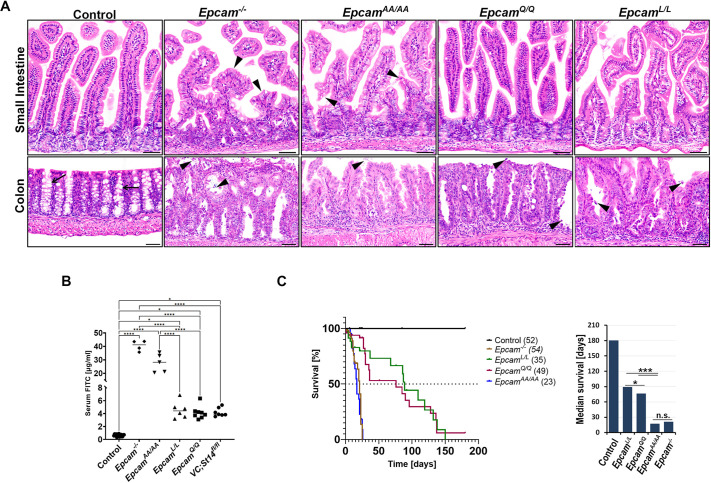
**Elimination of Arg80 leads to compromised intestinal development, growth retardation and shortened lifespan in mice.** (A) Representative images (from at least five mice per genotype) of H&E staining of small (top panels) and large (bottom panels) intestines from 21-day-old wildtype (control), *Epcam* null (*Epcam^−/−^*), *Epcam^AA/AA^*, *Epcam^Q/Q^* and *Epcam^L/L^* mice. Mice expressing EpCAM variants exhibited abnormal crypt architecture in the colon, associated with a loss of mucin-producing goblet cells (control, arrows) and increased shedding of intestinal epithelial cells (arrowheads). *Epcam^AA/AA^*, but not *Epcam^Q/Q^* and *Epcam^L/L^*, mice also present with irregular and tufted villi in the small intestine (arrowheads), similar to *Epcam^−/−^* mice. Scale bars: 50 µm. (B) Serum levels of FITC-dextran 3 h after oral gavage in 21-day-old wildtype (control, *n*=16), *Epcam* null (*Epcam^−/−^*, *n*=4), *Epcam^L/L^* (*n*=6), *Epcam^Q/Q^* (*n*=8), *Epcam^AA/AA^* (*n*=4) and *Vil-Cre;St14^fl/fl^* (*n*=7) mice. Complete loss of EpCAM or the expression of any of the variant lacking Arg80 increased intestinal permeability. Each data point represents a separate mouse. (C) Survival curve (left) and median survival (right) of wildtype (control, black), *Epcam^−/−^* (brown), *Epcam^L/L^* (green), *Epcam^Q/Q^* (red) and *Epcam^AA/AA^* (blue) mice. The numbers of mice of each genotype enrolled in the experiment are shown in parentheses. The expression of EpCAM variants was associated with significantly shortened lifespan. n.s., not significant; *P<0.05; ***P<0.001; ****P<0.0001.

At 3 weeks of age, *Epcam^AA/AA^* mice exhibited progressive, partial to complete alopecia and a severe growth retardation that was comparable with the phenotypes previously reported in *Epcam* null mice (*Epcam^−/−^*) (Fig. 2D) (Szabo et al., 2022). *Epcam^Q/Q^* mice presented with a ruffled coat and a slightly smaller size, whereas most *Epcam^L/L^* mice did not exhibit any obvious outward phenotype at this age (Fig. 2D). Histological analysis revealed changes in the epithelial architecture within both small and large intestines that included villous atrophy, indicated by shorter, irregular and tufted villi, which were particularly evident in the small intestines of *Epcam^AA/AA^* mice and were comparable in severity with those observed in *Epcam* null mice (*Epcam^−/−^*) (Fig. 3A). Small intestines of *Epcam^Q/Q^* or *Epcam^L/L^* mice appeared only minimally affected at this age (Fig. 3A). In contrast, all three knockin strains presented with prominent changes to the epithelia of their large intestines, which included increased epithelial erosion and shedding of epithelial cells into the lumen, loss of mucin-producing goblet cells, disorganization of the surface epithelium and lack of a well-organized crypt structure (Fig. 3A; Fig. S2A,B). Expression of several brush border-associated proteins, including the intestinal actin-binding protein villin (VIL1), the sodium-hydrogen antiporter NHE3 (also known as SLC9A3) and the principal glucose and galactose transporter GLUT2 (or SLC2A2), also appeared to be diminished in the enterocytes in small and large intestines of all *Epcam* knockin and *Epcam* knockout mice (Fig. S3). This is consistent with defective intestinal absorption previously reported in congenital diarrheal disorders, including CTE (Das et al., 2021).

Notch signaling has recently been implicated in the regulation of secretory cell differentiation in mouse intestines (Das et al., 2021). To determine whether the substantial loss of goblet cells in intestinal tissues from the *Epcam* knockout and all three knockin mice may be a result of altered Notch signaling, gene expression of *Notch1* and its downstream target *Hes1* was tested by quantitative real-time PCR (RT-PCR). No statistically significant changes were observed in the expression of either of the two genes (Fig. S2C).

Assessment of intestinal barrier function by oral administration of fluorescein isothiocyanate (FITC)-dextran revealed a strong increase in the permeability of intestinal epithelia in all three strains expressing cleavage-resistant EpCAM variants. In accordance with the histological findings presented above, the highest increase in FITC-dextran serum levels 3 h after gavage was observed in *Epcam^AA/AA^* mice. This barrier defect was comparable with that in *Epcam* null mice (43- and 63-fold increase, respectively, compared with wildtype controls), and it was considerably higher than the defect in *Epcam^Q/Q^* or *Epcam^L/L^* mice (6.4- and 6.8-fold increase, respectively, compared with wildtype controls) (Fig. 3B). Interestingly, barrier defects in *Epcam^Q/Q^* or *Epcam^L/L^* mice were quantitatively nearly identical to those observed in mice lacking intestinal matriptase (Fig. 3B). Mice expressing cleavage-resistant EpCAM variants also exhibited significantly shortened lifespan, with a median survival of 89 and 76 days for *Epcam^L/L^* and *Epcam^Q/Q^* mice, respectively, and only 17 days for *Epcam^AA/AA^* mice, compared with more than 180 days (experimental endpoint) for wildtype littermate controls (Fig. 3C). The median survival of mice lacking EpCAM expression was 21 days (Fig. 3C). Collectively, these results indicate that an intact cleavage site at Arg80 is indispensable for normal biological function of EpCAM during intestinal development.

### Expression of cleavage-resistant EpCAM does not prevent intestinal failure in *Spint2*-deficient mice

**Fig. 4.**
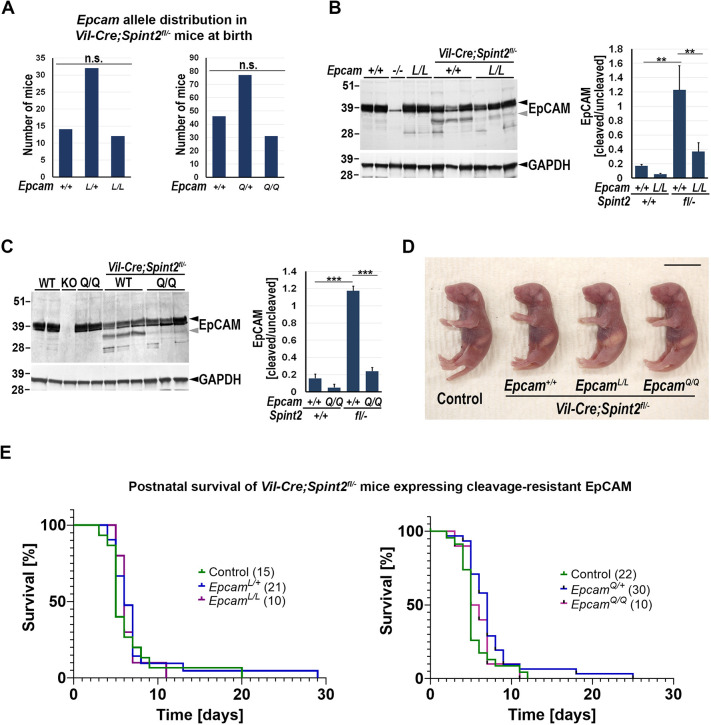
**Expression of R80L and R80Q variants suppresses EpCAM cleavage *in vivo* but fails to extend life of intestinal HAI-2-deficient mice.** (A) Distribution of *Epcam* genotypes among the HAI-2-deficient newborn offspring from *Vil-Cre^+/0^;Spint2^+/−^*;*Epcam^L/+^*×*Spint2^fl/+^*;*Epcam^L/+^* and *Vil-Cre^+/0^;Spint2^+/−^*;*Epcam^Q/+^*×*Spint2^fl/+^*;*Epcam^Q/+^* breeding pairs. Mice homozygous for R80L (*Epcam^L/L^*) and R80Q (*Epcam^Q/Q^*) mutations were born in the expected numbers. (B,C) Representative western blot (left, from two independent experiments) and the corresponding quantitative analysis of the ratio of the signal corresponding to cleaved versus uncleaved EpCAM (right) in intestines from 5-day-old wildtype mice (WT, *n*=4), *Epcam^−/−^* mice (KO, *n*=2), HAI-2-deficient mice (*Vil-Cre^+/0^;Spint2^fl/−^;Epcam^+/+^*, *n*=6) and HAI-2-deficient mice homozygous for R80L (B) or R80Q (C) mutations (*Vil-Cre^+/0^;Spint2^fl/−^*;*Epcam^L/L^* and *Vil-Cre^+/0^;Spint2^fl/−^*;*Epcam^Q/Q^* mice, respectively) (*n*=6 each). Quantification shows 80% (B) and 95% (C) reduction in cleavage of EpCAM caused by HAI-2-deficiency. (D) Representative image (from at least five mice per genotype) of the outward appearance of newborn wildtype (control) and HAI-2-deficient (*Vil-Cre^+/0^;Spint2^fl/−^*) mice expressing wildtype (*Epcam^+/+^*), R80L (*Epcam^L/L^*) or R80Q (*Epcam^Q/Q^*) EpCAM variants. None of the mice exhibited any obvious abnormality at birth. Scale bar: 1 cm. (E) Postnatal survival of HAI-2-deficient (*Vil-Cre^+/0^;Spint2^fl/−^*) mice homozygous for wildtype (control, green), and heterozygous (*Epcam^L/+^* and *Epcam^Q/+^*, blue) or homozygous (*Epcam^L/L^* and *Epcam^Q/Q^*, purple) for R80L (left) or R80Q (right) variants of EpCAM. The numbers of mice of each genotype enrolled in the experiment are shown in parentheses. The median postnatal survival of *Vil-Cre^+/0^;Spint2^fl/−^* mice was not affected by expression of cleavage-resistant EpCAM. n.s., not significant; **P<0.01, ***P<0.001.

To investigate whether excessive EpCAM cleavage and the resulting loss of EpCAM function does indeed drive the development of intestinal defects in the absence of HAI-2, intestinal epithelium-specific *Spint2* conditional knockout mice (*Vil-Cre^+/0^;Spint2^fl/−^*) were crossed to mice expressing cleavage-resistant EpCAM. The *Epcam^AA/AA^* strain that appears to be phenotypically equivalent to *Epcam* null mice (*Epcam^−/−^*) was at this point excluded from further study (Fig. 3A-C). Analysis of newborn offspring from *Vil-Cre^+/0^;Spint2^+/−^*;*Epcam^L/+^*×*Spint2^fl/+^*;*Epcam^L/+^* and *Vil-Cre^+/0^;Spint2^+/−^*;*Epcam^Q/+^*×*Spint2^fl/+^*;*Epcam^Q/+^* breeding pairs confirmed successful generation of *Vil-Cre^+/0^;Spint2^fl/−^*;*Epcam^L/L^* and *Vil-Cre^+/0^;Spint2^fl/−^*;*Epcam^Q/Q^* mice, respectively (Fig. 4A). Evaluation of the intestinal tissues by western blotting revealed a substantial reduction of EpCAM cleavage in both strains expressing cleavage-resistant EpCAM, compared with that in their *Vil-Cre^+/0^;Spint2^fl/−^*;*Epcam^+/+^* littermate controls (Fig. 4B,C). The reduction was particularly efficient in *Vil-Cre^+/0^;Spint2^fl/−^*;*Epcam^Q/Q^* mice, eliminating about 95% of the increase in cleavage caused by HAI-2-deficiency, compared with about 80% in *Vil-Cre^+/0^;Spint2^fl/−^*;*Epcam^L/L^* mice (Fig. 4B,C).

**Fig. 5.**
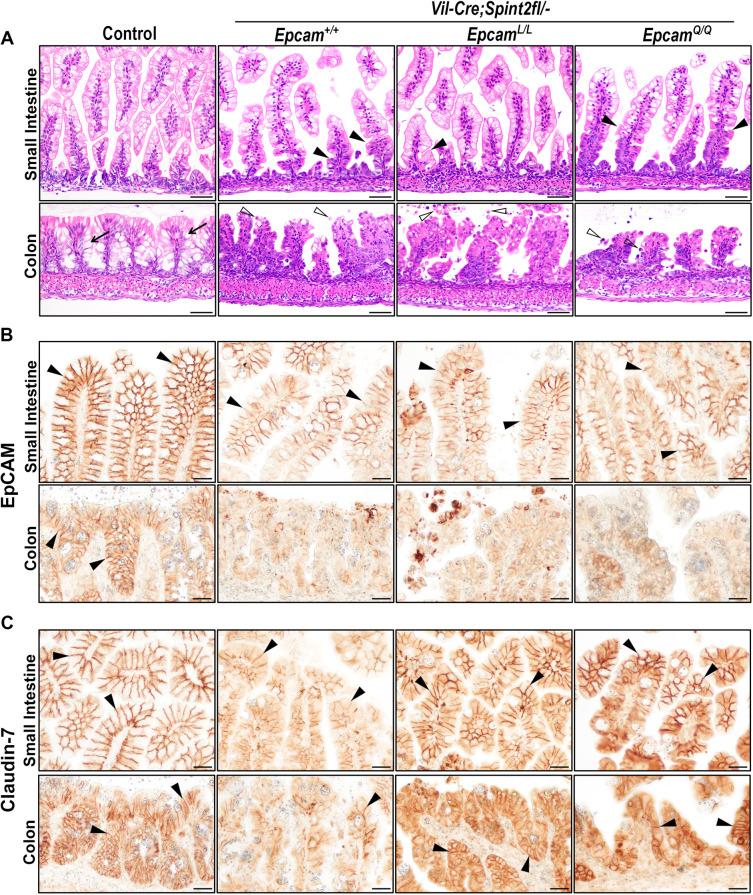
**Cleavage-resistant EpCAM does not prevent deterioration of intestinal epithelia in HAI-2-deficient mice.** (A-C) Representative images (from at least five mice per genotype) of H&E staining (A), and EpCAM (B) and claudin-7 (C) immunostaining of small (top panels) and large (bottom panels) intestines from 5 days old control (left), and HAI-2-deficient (*Vil-Cre^+/0^;Spint2^fl/−^*) mice expressing wildtype (*Epcam^+/+^*), R80L (*Epcam^L/L^*) or R80Q (*Epcam^Q/Q^*) EpCAM. Histological changes associated with a loss of HAI-2 function, including villous atrophy and tufting (A, arrowheads) in the small intestine, and increased epithelial erosion (A, open arrowheads), loss of mucin-producing goblet cells (A, arrows) and a loss of normal crypt structure in large intestines, were unaffected in mice expressing cleavage-resistant EpCAM. Similarly, loss of membrane-associated EpCAM expression (B, arrowheads) persisted in both the *Vil-Cre^+/0^;Spint2^fl/−^*;*Epcam^L/L^* and the *Vil-Cre^+/0^;Spint2^fl/−^*;*Epcam^Q/Q^* mice. In contrast, overall expression and membrane localization of claudin-7 (C, arrowheads) were largely restored in HAI-2-deficient mice expressing R80L or R80Q EpCAM. Scale bars: 50 µm (A); 25 µm (B,C).

*Vil-Cre^+/0^;Spint2^fl/−^*;*Epcam^L/L^* and *Vil-Cre^+/0^;Spint2^fl/−^*;*Epcam^Q/Q^* mice did not display any obvious outward phenotype at birth and were similar in size and appearance to their wildtype or *Vil-Cre^+/0^;Spint2^fl/−^*;*Epcam^+/+^* littermate controls (Fig. 4D). However, expression of R80L or R80Q EpCAM failed to prevent any of the defects in intestinal development or to extend the overall survival in mice lacking HAI-2. *Vil-Cre^+/0^;Spint2^fl/−^* mice typically died within 10 days after birth (median survival of 5-7 days), irrespective of whether they were wildtype, heterozygous or homozygous for either of the cleavage-resistant EpCAM variants (Fig. 4E). Histological examination of the intestinal tissues from 5-day-old mice revealed that the expression of R80L or R80Q EpCAM failed to prevent deterioration of epithelia in small or large intestines induced by the loss of HAI-2. The overall histological appearance, the reduction in the number of goblet cells in both small and large intestines, and the presence of tufts were all unaffected in *Vil-Cre^+/0^;Spint2^fl/−^*;*Epcam^L/L^* and *Vil-Cre^+/0^;Spint2^fl/−^*;*Epcam^Q/Q^* mice, compared with their *Vil-Cre^+/0^;Spint2^fl/−^* littermates that expressed wildtype EpCAM (Fig. 5A; Fig. S4). Additionally, loss of overall expression and membrane localization of EpCAM observed in the intestines of mice lacking HAI-2 also remained unaffected in mice expressing cleavage-resistant variants (Fig. 5B). In contrast, the expression of claudin-7, which is also substantially diminished in the intestines of *Vil-Cre^+/0^;Spint2^fl/−^* mice, was largely restored in mice homozygous for genes encoding either R80L or R80Q EpCAM, although its cellular localization did not appear to be as strongly membrane-associated as in the epithelia of wildtype littermate controls (Fig. 5C). In summary, these result show that the early-onset intestinal defects in HAI-2-deficient mice are unaffected by the suppression of EpCAM proteolysis in mice expressing cleavage-resistant EpCAM proteins.

### Elimination of matriptase prevents early-onset intestinal defects in *Spint2*-deficient mice expressing cleavage-resistant EpCAM

We have previously demonstrated that inactivation of matriptase led to a significant suppression of the intestinal defects and early postnatal lethality in HAI-2 null mice, although it was not able to completely prevent intestinal dysfunction, and HAI-2/matriptase double-deficient mice typically died around 3 weeks of age (Szabo et al., 2019). Consistently, genetic ablation of intestinal matriptase led to a substantial increase in the overall lifespan in *Vil-Cre^+/0^;Spint2^fl/−^* mice (median survival of 23 days, compared with 5 days for matriptase-expressing *Vil-Cre^+/0^;Spint2^fl/−^* mice, Fig. S5A) but did not completely restore normal growth and the histological appearance of small and large intestines (Fig. S5B,C).

**Fig. 6.**
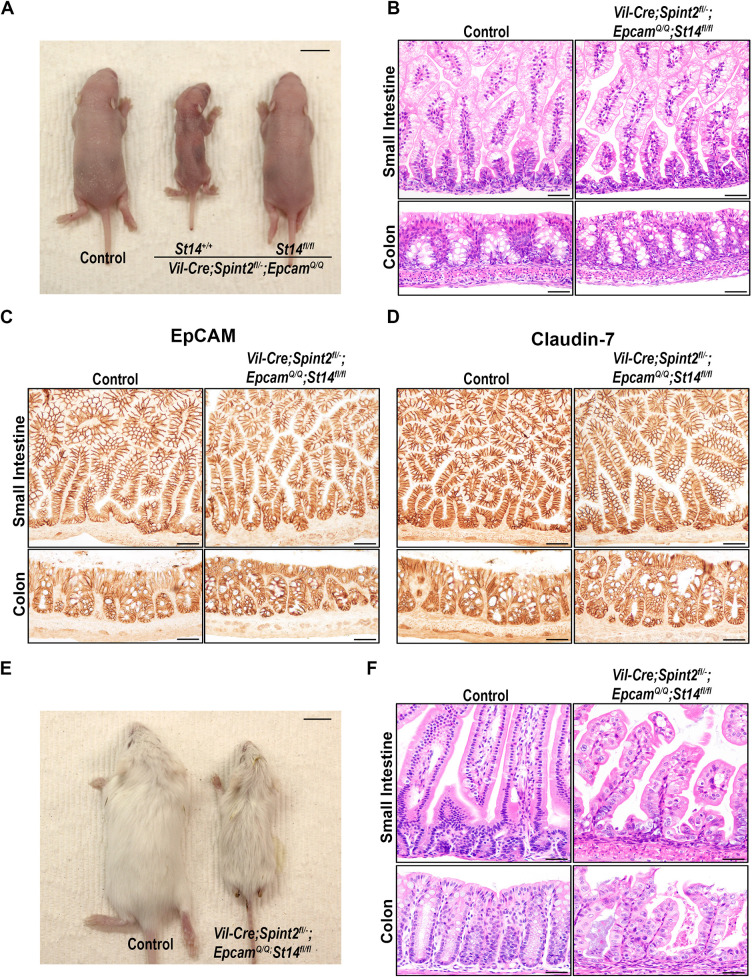
**Intestinal defects in HAI-2-deficient mice expressing cleavage-resistant EpCAM are caused by matriptase.** (A) Representative image (from at least five mice per genotype) of the overall appearance of 5-day-old matriptase-expressing (*St14^+/+^*) and intestinal matriptase-deficient (*St14^fl/fl^*) *Vil-Cre^+/0^;Spint2^fl/−^*;*Epcam^Q/Q^* mice, and their wildtype littermates (control). Inactivation of matriptase restored normal growth in mice lacking HAI-2 even when expressing cleavage-resistant EpCAM. (B-D) Representative images (from at least five mice per genotype) of H&E staining (B), and EpCAM (C) and claudin-7 (D) immunostaining of small (top panels) and large (bottom panels) intestines from 5-day-old control mice (left) and *Vil-Cre^+/0^;Spint2^fl/−^*;*Epcam^Q/Q^* mice lacking intestinal matriptase (*Vil-Cre^+/0^;Spint2^fl/−^*;*Epcam^Q/Q^;St14^fl/fl^*, right). Inactivation of matriptase restored normal histological appearance, and EpCAM and claudin-7 expression and cellular localization were all indistinguishable from those in wildtype littermate controls. (E,F) Representative image (four mice per genotype) of the overall appearance (E) and the intestinal histology (H&E) (F) of 21-day-old control and *Vil-Cre^+/0^;Spint2^fl/−^*;*Epcam^Q/Q^;St14^fl/fl^* mice. Mice with a combined HAI-2/matriptase deficiency presented with smaller size and abnormal histological morphology. Scale bars: 1 cm (A,E); 50 µm (B-D,F).

As we have previously shown that the development of intestinal defects and the increased cleavage of EpCAM in mice lacking HAI-2 are largely dependent on the activity of matriptase (Szabo et al., 2019) (Fig. 1B), We next tested whether intestinal development in *Spint2*-deficient mice expressing cleavage-resistant EpCAM could still be normalized by elimination of matriptase activity. Thus, we genetically inactivated intestinal matriptase by crossing *Vil-Cre^+/0^;Spint2^fl/−^*;*Epcam^Q/Q^* mice to mice carrying a matriptase conditional knockout allele (*St14^fl/fl^*). Strikingly, *Vil-Cre^+/0^;Spint2^fl/−^*;*Epcam^Q/Q^;St14^fl/fl^* mice did not exhibit any obvious outward phenotype within the first week of their life, were comparable in size and appearance with their wildtype littermate controls, and were considerably larger than their *Vil-Cre^+/0^;Spint2^fl/−^*;*Epcam^L/L^* or *Vil-Cre^+/0^;Spint2^fl/−^*;*Epcam^Q/Q^* littermates that expressed functional matriptase (Fig. 6A). Elimination of matriptase also completely normalized the histological appearance of epithelia in both small and large intestines of 5-day-old mice (Fig. 6B, compare with Fig. 5A). Similarly, the expression and cell-surface localization of both EpCAM and claudin-7 proteins were restored in *Vil-Cre^+/0^;Spint2^fl/−^*;*Epcam^Q/Q^* mice lacking intestinal matriptase (Fig. 6C,D, compare with Fig. 5B,C, respectively). Western blot analysis of intestinal tissues from 5-day-old *Vil-Cre^+/0^;Spint2^fl/−^*;*Epcam^Q/Q^;St14^fl/fl^* and their matriptase-expressing *Vil-Cre^+/0^;Spint2^fl/−^*;*Epcam^Q/Q^* counterparts did not reveal significant differences in the proteolytic processing of EpCAM, indicating that the phenotypic rescue is not cleavage related (Fig. S5D). These results show that matriptase drives the loss of EpCAM expression and the development of the early-onset intestinal defects in HAI-2-deficient mice, even when its ability to cleave EpCAM is disrupted.

*Vil-Cre^+/0^;Spint2^fl/−^*;*Epcam^Q/Q^;St14^fl/fl^* mice exhibited slower growth and were visibly smaller in their second and third week of life (Fig. 6E). At 3 weeks of age, histological changes were detected in both small and large intestines of these mice, and included villous atrophy, tufting, and general loss of crypt structure and disorganized surface epithelium in the colon (Fig. 6F). All *Vil-Cre^+/0^;Spint2^fl/−^*;*Epcam^Q/Q^;St14^fl/fl^* mice presented with severe diarrhea and had to be euthanized at the end of the third week of life due to health concerns. Interestingly, the outward appearance, lifespan and histological phenotype of *Vil-Cre^+/0^;Spint2^fl/−^*;*Epcam^Q/Q^;St14^fl/fl^* mice closely resembled those of *Vil-Cre^+/0^;Spint2^fl/−^*;*Epcam^+/+^;St14^fl/fl^* mice (Fig. 6E,F, compare with Fig. S5A-C), further indicating that EpCAM cleavage does not play a crucial role in intestinal dysfunction caused by HAI-2-deficiency.

## DISCUSSION

Loss of function of the epithelial cell adhesion protein, EpCAM, is the underlying cause of intestinal dysfunction in most individuals with CTE (Sivagnanam et al., 2008; Salomon et al., 2014; AlMahamed and Hammo, 2017; Pathak et al., 2019). However, the discovery of mutations in *SPINT2*, encoding the serine protease inhibitor HAI-2, in individuals with sCTE revealed a crucial role for cell-surface proteolysis in the etiology of this disease (Heinz-Erian et al., 2009; Salomon et al., 2014). Consequently, it was proposed that an excessive cleavage of EpCAM by matriptase, or a related trypsin-like serine protease, resulting from lack of inhibition by HAI-2, is the primary cause of the compromised epithelial barrier and intestinal failure in *SPINT2*-deficient individuals (Wu et al., 2017, 2020; Holt-Danborg et al., 2019). Indeed, the subsequent generation of *Spint2*-deficient mouse models confirmed that the loss of HAI-2 leads to an increased activation of intestinal matriptase (Szabo et al., 2019) Importantly, chemical inhibition, shRNA-mediated knockdown or genetic inactivation of matriptase restored EpCAM expression and prevented loss of epithelial integrity and early postnatal demise caused by HAI-2 deficiency in intestinal organoid culture and in live mice (Szabo et al., 2019; Kawaguchi et al., 2019).

Here, we show that the absence of functional HAI-2 does indeed lead to a substantial increase in proteolysis of intestinal EpCAM *in vivo* that is nearly completely dependent on matriptase activity. However, our data indicate that contrary to the proposed model, excessive cleavage of EpCAM is not crucially involved in the progressive intestinal failure observed in mice lacking intestinal HAI-2. First, expression of cleavage-resistant variants of EpCAM failed to protect *Spint2*-deficient mice from the early-onset intestinal dysfunction. Thus, elimination of the verified matriptase cleavage site by replacing the crucial Arg80 with leucine (R80L) or glutamine (R80Q) prevented 80 and 95%, respectively, of EpCAM cleavage induced by loss of HAI-2 *in vivo*, yet it did not diminish any of the macroscopic or histological defects caused by HAI-2 deficiency. It also had no impact on the overall survival of *Vil-Cre^+/0^;Spint2^fl/−^* mice. Second, as the development of the CTE-like phenotype in HAI-2-deficient mice was almost entirely dependent on matriptase activity, one would expect that if the intestinal demise was caused by cleavage of EpCAM, then elimination of matriptase function should have limited or no impact on HAI-2-deficient mice expressing cleavage-resistant EpCAM variants. However, genetic inactivation of intestinal matriptase completely counteracted the effects of HAI-2-deficiency on the early postnatal development in *Vil-Cre^+/0^;Spint2^fl/−^*;*Epcam^Q/Q^;St14^fl/fl^* mice, despite the inability of the protease to induce a substantial increase in cleavage of R80Q EpCAM *in vivo*.

It should be noted that we detected low levels of EpCAM cleavage in the intestines from matriptase-deficient (*Vil-Cre^+/0^;Spint2^fl/−^;St14^fl/fl^*) mice (Fig. 1B). This is most likely a result of an incomplete recombination of one or both *St14* flox alleles. Indeed, in our initial characterization of *Vil-Cre^+/0^;St14^fl/fl^* strain, we reported a very low, but still detectable, level of the wildtype *St14* transcript in both small and large intestines (Kosa et al., 2012). Alternatively, the residual cleavage may indicate the presence of another EpCAM-processing protease. This would be consistent with the reports by Pavšič et al. (2014) and Schon et al. (1993) that show an efficient *in vitro* cleavage of EpCAM by a variety of proteases, including cathepsins L and K, plasmin, thrombin, trypsin and chymotrypsin. Although our data clearly identify matriptase as the major EpCAM-processing enzyme *in vivo*, contribution from other proteases cannot be excluded, especially under permissive conditions caused by the absence of HAI-2.

**Fig. 7.**
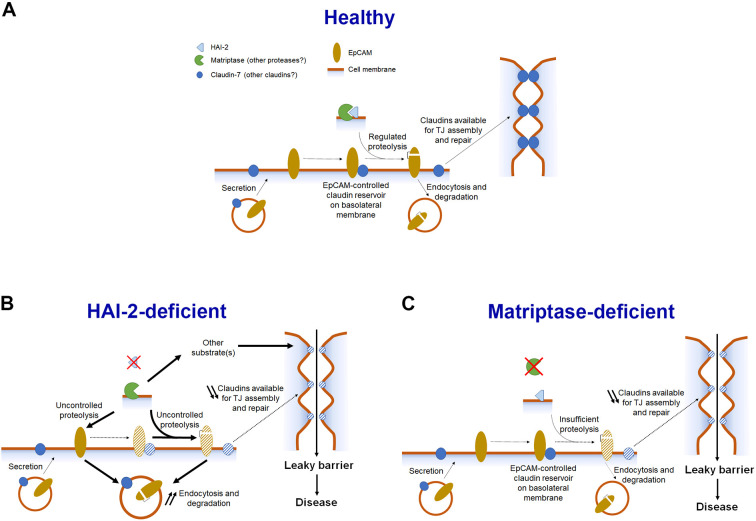
**Proposed interaction between HAI-2, matriptase and EpCAM during intestinal development and homeostasis.** (A-C) Schematic representation of the proposed roles of EpCAM, HAI-2 and matriptase in healthy (A), HAI-2-deficient (B) and matriptase-deficient (C) intestines. (A) In the healthy tissue, EpCAM binds and stabilizes claudin-7, and possibly other claudins, on the basolateral surface of epithelial cells. When needed, claudin(s) are released from the complex by regulated cleavage of EpCAM by matriptase, thus becoming available for the tight junction (TJ) assembly and repair. (B) In the absence of HAI-2, increase in matriptase activity leads to an uncontrolled cleavage of EpCAM, resulting in its endocytosis and degradation. This depletes the available pool of claudin-7 at the cell surface, diminishing its ability to contribute to TJ assembly and repair. However, as normal intestinal development is not restored in HAI-2-deficient mice expressing cleavage-resistant EpCAM, increased cleavage of other substrates by matriptase, and possibly by other trypsin-like serine proteases, is expected to contribute to the intestinal demise in these mice. (C) Finally, in the absence of matriptase (or upon the expression of cleavage-resistant EpCAM), the formation of EpCAM/claudin complexes is not affected, but the diminished ability to cleave EpCAM limits the amount of claudin(s) that can be released from the complex. This again diminishes the ability of the epithelium to assemble and/or repair the TJs.

Given the well-established association between the loss of EpCAM function and the development of intestinal insufficiency in CTE individuals and *Epcam*-deficient mouse models, and in view of previous work linking the loss of HAI-2 and the resulting increase in matriptase activity to regulation of EpCAM stability and function, this finding is unexpected. It is important to point out that we cannot formally exclude the possibility that the loss of HAI-2 can compromise EpCAM function by mechanisms that are not directly dependent on its cleavage. The fact that, despite a nearly complete suppression of cleavage, EpCAM expression and membrane localization was not restored in HAI-2-deficient mice expressing R80L and R80Q variants could indicate that the decline in EpCAM protein stability and cellular distribution is at least partially independent of its proteolytic processing (Figs 4B,C and 5B). As both the expression and localization of EpCAM were normalized in the absence of matriptase, the mechanism would likely include another, yet unknown matriptase substrate, which, upon cleavage, could trigger internalization and/or shedding of the EpCAM protein, in turn leading to the deterioration of intestinal function. However, HAI-2 might also facilitate intestinal development by a mechanism that is independent of EpCAM cleavage, and the increase in proteolytic processing of EpCAM that is observed in HAI-2-deficent intestines may simply be a consequence of histological and functional changes caused by unchecked activity of matriptase and possibly other proteases regulated by HAI-2 (Fig. 7). Therefore, it is plausible that despite the similarities in histological appearance and in clinical outcome between the EpCAM- and HAI-2-deficient CTE individuals, and between the corresponding mouse models, the two proteins may regulate intestinal development by molecular mechanisms that are at least partially independent of each other. Indeed, HAI-2 has recently been shown to regulate hatching gland morphogenesis in zebrafish independently of EpCAM and at least partially via the epithelial cell adhesion protein E-cadherin (Hatzold et al., 2021). However, it should be noted that in the zebrafish model, HAI-2 also appeared to act independently of matriptase and, to our knowledge, E-cadherin has not been reported to be proteolytically regulated by any of the HAI-2 target proteases.

Processing by extracellular proteases is generally assumed to impede normal biological function of EpCAM. Indeed, cleavage within the TY loop that contains the dibasic site Arg80-Arg81 was shown to prevent EpCAM dimerization, to decrease its ability to associate with claudin-7 and to target it for lysosomal degradation (Pavšič et al., 2014; Wu et al., 2017). In this respect, our finding that mice expressing cleavage-resistant EpCAM exhibit late-onset intestinal dysfunction and significantly shortened lifespan, indicative of partial (*Epcam^L/L^* and *Epcam^Q/Q^*) to complete (*Epcam^AA/AA^*) loss of function, is surprising. However, it is in line with a recent study by Higashi and colleagues proposing that EpCAM proteolysis is, in fact, crucial for the dissociation of EpCAM/claudin-7 complexes and subsequent incorporation of claudin-7 into forming or damaged tight junctions (Higashi et al., 2023). EpCAM cleavage may therefore play a particularly important role in rapidly renewing epithelia of small and large intestines to facilitate continuous repair of epithelial barrier compromised by the loss of cells at the extrusion zones. The apparent normalization of claudin-7 expression and its increased accumulation at the basolateral surface in HAI-2-deficient intestinal epithelia expressing cleavage-resistant EpCAM are seemingly consistent with this model (Fig. 5C). The observed differences in the severity of the phenotypes between the *Epcam* knockin strains could therefore also be explained by an incomplete suppression of *in vivo* cleavage in *Epcam^L/L^* and *Epcam^Q/Q^* mice compared with a more efficient suppression in *Epcam^AA/AA^* mice. Indeed, whereas the R80L and R80Q EpCAM variants appeared to be completely resistant to matriptase-mediated cleavage in HEK293T cells, the cleaved form was clearly detected in both the *Vil-Cre^+/0^;Spint2^fl/−^*;*Epcam^Q/Q^* and the *Vil-Cre^+/0^;Spint2^fl/−^*;*Epcam^L/L^* intestines. This would indicate that EpCAM processing in the cell expression system may not fully reflect the *in vivo* conditions. Whether this residual cleavage is the result of a proteolysis at the adjacent Arg81, mediated by matriptase, a similar trypsin-like protease or another protease capable of cleaving at the modified cleavage site, remains to be determined.

Interestingly, mice expressing cleavage-resistant EpCAM variants bear a remarkable phenotypic similarity to previously described intestinal matriptase-deficient mice. *Vil-Cre^+/0^;St14^fl/fl^* mice present with comparable intestinal barrier defects, histological changes affecting predominantly the colon, watery diarrhea and a reduced lifespan of 2-4 months (Fig. S5) (Kosa et al., 2012). This indicates the intriguing possibility that the intestinal defects observed in matriptase-deficient mice may in fact result from an inefficient cleavage of EpCAM, which leads to a diminished ability to release claudins and repair damaged barrier (Fig. 7). If true, this would identify EpCAM as a long-sought-after physiological substrate of matriptase, at least in the context of intestinal development and homeostasis.

Although the suppression of EpCAM cleavage affects the development and function of both the small and large intestines, histological changes are primarily observed in colon, as evidenced by a severe deterioration of normal crypt and surface epithelium architecture, near complete loss of goblet cells and notable erosion of colonic epithelium, compared with relatively mild changes observed in the small intestines (Fig. 3A). This likely reflects a higher degree of mechanical abrasion and an increased exposure of the colonic epithelium to gut microbiome, which are expected to further escalate the damage caused by an impaired barrier repair owing to the ineffective cleavage of EpCAM. Indeed, we have previously reported that an elimination of the gut microbiome in matriptase-deficient mice strongly suppressed inflammation and nearly completely normalized the histological appearance of colonic tissue (Kosa et al., 2012).

In summary, we show that, in contrast to the previously proposed model, excessive cleavage of EpCAM does not appear to be crucially involved in the etiology of intestinal malfunction in HAI-2-deficient mice and that EpCAM processing is in fact crucial for its normal biological function. By extension, these data indicate that the molecular etiology of sCTE may differ from the etiology of conventional CTE driven by the mutations in *EPCAM* gene, and that the development of therapies aimed at inhibiting or blocking EpCAM cleavage may be of limited use, if not detrimental, in preventing the loss of intestinal function in *SPINT2*-deficient individuals.

## MATERIALS AND METHODS

### Mice

Experiments involving mice were performed in an Association for Assessment and Accreditation of Laboratory Animal Care International-accredited vivarium following Institutional Guidelines and Standard Operating Procedures, and were approved by the National Institute of Dental and Craniofacial Research (NIDCR) Institutional Animal Care and Use Committee. Mice carrying *Spint2* full and conditional knockout (*Spint2^−^* and *Spint2^fl^*), matriptase conditional knockout (*St14^fl^*), *Epcam* full knockout (*Epcam^−^*) and intestinal-specific expression of Cre recombinase (*Vil-Cre^+^*^/0^) alleles have all been described previously (Szabo et al., 2009, 2022; List et al., 2009; Kawaguchi et al., 2019).

To generate knockin mice expressing R80L, R80Q and R80A/R81A variants of EpCAM, two guide RNAs targeting exon 4 of the *Epcam* gene were designed using the CHOP-CHOP CRISPR guide RNA design tool (http://chopchop.cbu.uib.no/; Labun et al., 2019) and synthesized by Horizon Discovery Biosciences (Cambridge, UK) (see Table S1 for guide sequences). 200 nt single-strand DNAs (ssDNAs) carrying the desired mutations in codon 80 or codons 80 and 81 of the *Epcam* gene were used as donor templates (synthesized by Integrated DNA Technologies, Coralville, IA, USA; see Table S2 for sequences). 25 ng/μl of each guide RNA, 10 ng/μl of the ssDNA donor and 100 ng/μl recombinant *Sp*Cas9 protein (PNA Bio) were pre-mixed in 10 mM Tris/HCl, pH 7.4, 0.1 mM EDTA buffer, and microinjected into the male pronucleus of FVB/NJ zygotes, followed by implantation into pseudopregnant female mice. All founders were screened for changes in the targeted regions by PCR amplification followed by DNA sequencing (see Table S1 for primer sequences; 35 cycles at 94°C/56°C/72°C, 30 s per step). Mice carrying the R80L (*Epcam^L/+^*), R80Q (*Epcam^Q/+^*) and R80A/R81A (*Epcam^AA/+^*) mutations were bred to FVB/NJ wildtype mice to establish stable mouse lines and then intercrossed with mice carrying the appropriate *Spint2*, *St14* and *Cre* alleles to produce experimental animals. All studies used mice of mixed 129S6/Sv;NIH BlackSwiss;FVB/NJ;C57Bl/6J genetic background and were littermate controlled. Mice were genotyped by PCR using ear or tail clips of newborn to 2-week-old mice as described elsewhere (see Table S1 for primer sequences) (Szabo et al., 2009, 2022; List et al., 2009; Kawaguchi et al., 2019).

### Cell culture and transfection

HEK293T human embryonic kidney cells were obtained from American Type Culture Collection. Cells were routinely maintained in high-glucose Dulbecco's modified Eagle medium (DMEM) supplemented with 10% fetal bovine serum (FBS) (both Life Technologies) at 37°C with 5% CO_2_. For transfection, 50,000 cells/well were seeded on a 12-well plate and grown in DMEM/FBS until reaching 60-80% confluency. The cells were then transfected with 0.5 µg pCMV6-AC plasmid DNA expressing human EpCAM variants (all synthesized by Blue Heron Biotech) with or without 0.3 µg of plasmid DNA expressing full-length human matriptase (Oberst et al., 2005) using the Lipofectamine 3000 transfection kit (Invitrogen) following the manufacturer's instructions. Alternatively, the cells were transfected with pCMV6-AC-HA and pCMV6-AC-Myc plasmid DNAs to express C-terminally HA- and Myc-tagged human EpCAM variant proteins (all plasmids synthesized by Blue Heron Biotech). Cells were washed with serum-free DMEM 48 h after transfection, lysed in 1 ml of ice-cold 50 mM Tris/HCl, pH 8.0, 1% NP-40, 500 mM NaCl buffer and incubated on ice for 10 min. The lysates were then centrifuged at 20,000 ***g*** for 10 min at 4°C to remove the cellular debris and stored at −20°C until further use.

### Immunoprecipitation

For immunoprecipitation, 0.5 ml of cleared cell lysates (see above) were diluted with 0.5 ml of 50 mM Tris/HCl, pH 8.0, 1% NP-40, 500 mM NaCl buffer and pre-incubated with 50 µl Pierce Protein A/G Plus Agarose beads (Thermo Fisher Scientific) for 30 min at 4°C with gentle agitation. The samples were spun at 1000 ***g*** for 5 min to remove the beads, and the supernatant was incubated with 3 µg rabbit anti-HA tag (3724, Cell Signaling Technology), mouse anti-Myc tag (2276, Cell Signaling Technology) or goat anti-EpCAM (AF960, R&D Systems) antibody and 50 µl of Pierce Protein A/G Plus Agarose beads for 16 h at 4°C. The samples were spun at 1000 ***g*** for 1 min, the supernatant was removed, and the beads were washed three times with 1 ml ice-cold 50 mM Tris/HCl, pH 8.0, 1% NP-40, 500 mM NaCl buffer. The beads were then mixed with 30 µl of 1× SDS loading buffer (Invitrogen) with 0.25 M β-mercaptoethanol (Sigma-Aldrich), incubated for 5 min at 99°C, and cooled on ice for 2 min. The samples were spun at 1000 ***g*** for 1 min and the released proteins were resolved by SDS-PAGE (4-12% polyacrylamide gel) and analyzed by western blotting as described below. To directly compare the efficacy of the dimerization of the EpCAM variants, 1:3 serial dilutions corresponding to 5.0, 1.67, 0.56, and 0.19 µl of anti-HA immunoprecipitation eluates were resolved by SDS-PAGE and the amounts of Myc-tagged EpCAM protein were visualized by anti-Myc immunoblotting.

### Western blotting

Small and large intestines were collected at postnatal day 5 or 20, snap-frozen in liquid nitrogen, and stored at −80°C until further use. For western blot analysis, the tissues were homogenized in 2% SDS and 10% glycerol in 62.5 mM Tris/Cl pH 6.8 containing protease inhibitor cocktail (Sigma-Aldrich). The lysates were cleared by centrifugation at 16,000 ***g*** for 10 min at 4°C to remove the tissue debris, and the protein concentration in the supernatant was determined by BCA assay (Pierce, Rockford, IL, USA). For western blot detection, 50 µg of total protein was mixed with 4× SDS sample buffer (NuPAGE, Invitrogen) containing 7% β-mercaptoethanol, boiled for 5 min at 99°C, and run on 4–12% BisTris NuPAGE gels using 1× MOPS running buffer (both Invitrogen). The separated proteins were transferred to PVDF membranes (0.2 μm, Invitrogen) and blocked with 5% nonfat dry milk in TBS containing 0.2% Tween 20 (TBS-T). Membranes were incubated with primary antibody overnight at 4°C, followed by incubation with secondary antibody conjugated to alkaline phosphatase for 1.5 h at room temperature (see Table S3 for information on antibodies). Alkaline phosphatase activity was visualized using nitro-blue tetrazolium and 5-bromo-4-chloro-3'-indolylphosphate substrates (Sigma-Aldrich). Data shown are representative of at least two independent western blotting experiments. Each lane represents a different mouse. Where indicated, band intensities were quantified using ImageJ (National Institutes of Health, Bethesda, MD, USA; version 1.53c).

### Histological analysis

Mice were euthanized on postnatal days 5 or 20, and intestinal tissues were extracted and immediately fixed in aqueous-buffered zinc formalin fixative (Z-Fix, Anatech Ltd.) for 24 h at room temperature prior to paraffin embedding and sectioning (Histoserv Inc.); 5-µm-thick sections were stained with Hematoxylin & Eosin (H&E) or Alcian Blue/Periodic Acid Shiff (AB/PAS) (all performed by Histoserv Inc.), or used for immunohistochemistry as described below. All histological images are representative of at least five independent mice per genotype.

### Immunohistochemistry

Five-micrometer-thick sections from formalin-fixed, paraffin-embedded mouse tissues were immunostained after antigen retrieval by incubation for 20 min at 100°C in 0.01 M sodium citrate buffer, pH 6.0, essentially as described previously (Szabo et al., 2016). Briefly, the sections were blocked with 3% bovine serum albumin (Fraction V, MP Biomedicals, Solon, OH, USA) in PBS and incubated overnight at 4°C with primary antibodies (see Table S3 for further information on antibodies). Bound antibodies were visualized using biotin-conjugated anti-rabbit or anti-goat secondary antibodies (BA-1000 and BA-9500, respectively, Vector Laboratories) and a Vectastain ABC Kit (Vector Laboratories), using 3,3′-diaminobenzidine as the substrate (Sigma-Aldrich). All microscopy images were acquired on Olympus BX43 microscope using an Olympus DP74 digital camera with cellSens Entry system (all Olympus).

### Intestinal permeability assay

*In vivo* intestinal permeability was determined as described previously (List et al., 2009). In brief, 3-week-old mice were gavaged with 10 µl/g body weight of a solution of 22 mg/ml FITC-dextran (molecular mass 4 kDa, Millipore Sigma) in PBS, pH 7.4. Mice were euthanized by CO_2_ inhalation and blood was collected from the inferior vena cava 3 h after the gavage, and plasma was collected by centrifugation at 8000 ***g*** for 10 min at 4°C. 25 µl of plasma in 75 μl of PBS was added to a 96-well plate. The concentration of fluorescein was determined using a Synergy NEO microplate reader (Biotek Instruments) with an excitation wavelength of 485 nm and an emission wavelength of 535 nm, using serially diluted samples of the tracer in 25% goat serum as a standard.

### RNA preparation and quantitative RT-PCR

Small and large intestines collected from postnatal day 1 mice were homogenized in TRIzol reagent (Life Technologies) and total RNA was extracted according to the manufacturer's instructions. RNA (2 µg) was reverse transcribed with oligo-dT primers using the RevertAid H Minus First Strand cDNA Synthesis Kit (Thermo Fisher Scientific). Real-time PCR was conducted on 1 µl of cDNA template using iQ SYBR Green Supermix (Bio-Rad Laboratories) and 7500 Real-Time PCR System with 7500 Software v2.3 (Applied Biosystems). The primer sets used for cDNA amplification are listed in Table S1. The expression of each gene was normalized to the expression of *Gapdh* using the ΔΔCt method. The assay was performed in duplicate. Results shown are the means and standard deviations from three independent mice per genotype.

### Statistics

The quantification of protein signals on western blots was statistically analyzed using two-sample, two-tailed, unpaired Student's *t*-test when comparing two groups or a one-way ANOVA with Tukey multiple comparison post-hoc test to compare three or more groups. Each lane represents a sample from a separate animal. Only signals present on the same membrane were statistically compared.

Allele distribution at birth was evaluated using χ^2^ analysis of the observed versus the expected numbers of *Epcam^+/+^*, *Epcam^+/−^* and *Epcam^Ki/Ki^* (*Epcam^L/L^* or *Epcam^Q/Q^*) mice among *Vil-Cre;Spint2^fl/−^* offspring from *Vil-Cre;Spint2^fl/−^;Epcam^Ki/+^*×*Spint2^+/−^;Epcam^Ki/+^* parents. Postnatal survival was analyzed using Mantel–Cox log-rank test.

Plasma concentrations of FITC-dextran as a function of intestinal transepithelial permeability were determined for at least five independent mice per genotype and statistically evaluated using a one-way ANOVA with Tukey multiple comparison post-hoc test.

All statistical analysis was performed using GraphPad Prism software (GraphPad Prism ver.9.0.0, GraphPad Software).

## Supplementary Material

10.1242/develop.201801_sup1 Supplementary information
