# Supplementary material for: Early-onset tufting enteropathy in HAI-2-deficient mice is independent of matriptase-mediated cleavage of EpCAM
Source: Development. 2023 Aug 31;150(17):dev201801. doi: 10.1242/dev.201801 (PMC10482385; doi:10.1242/dev.201801)
Supplement: Supplementary information [file develop-150-201801-s1.pdf]

## Figure S1

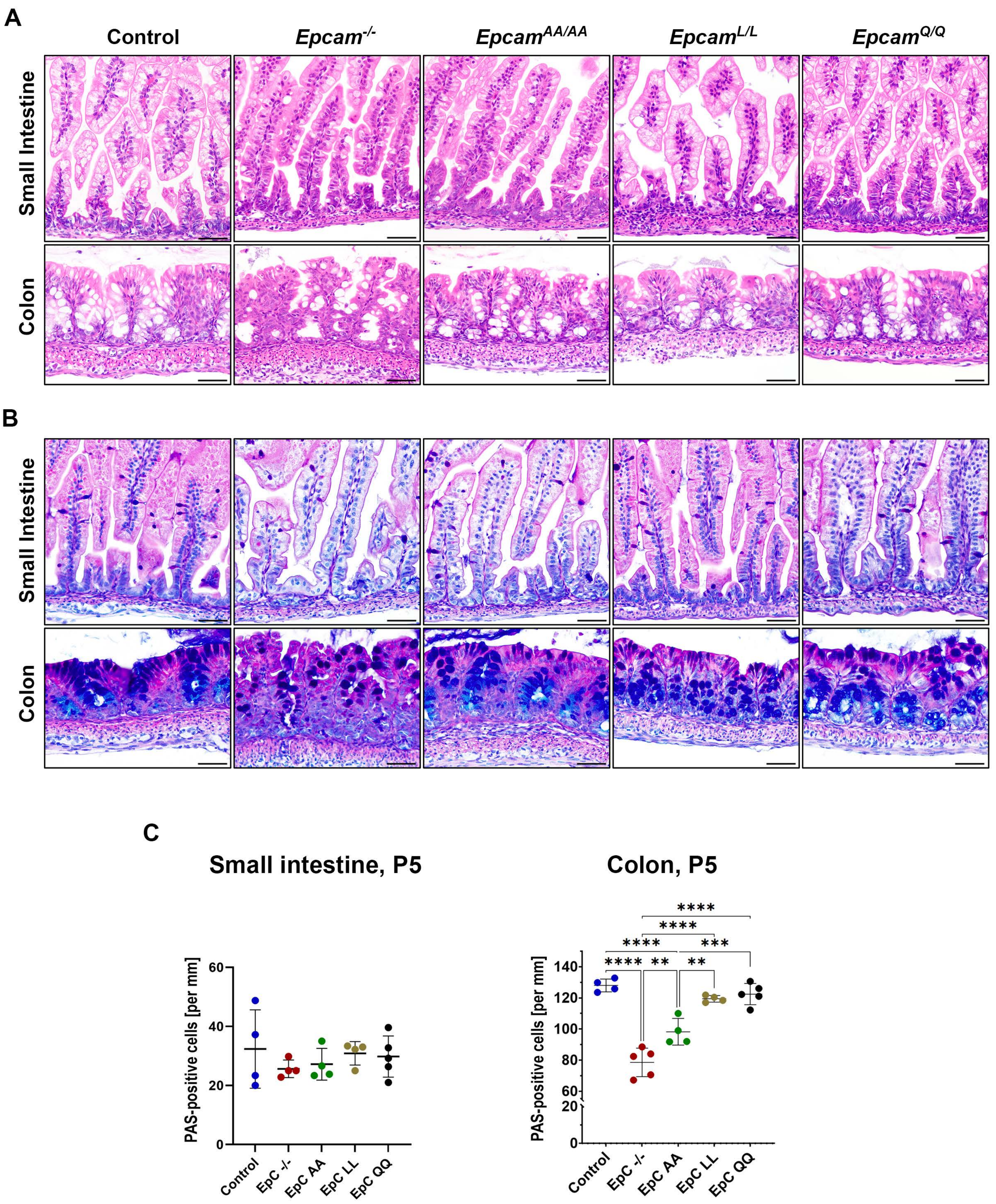


**Fig. S1. Expression of R80A/R81A, but not R80L and R80Q variants, affects intestinal morphology in the first week of life.** (**A, B**). Representative images (from at least 5 mice per genotype) of H&E **(A)** and Alcian blue/PAS **(B)** staining of small (top panels) and large (bottom panels) intestines from 5 days old wildtype (Control), EpCAM null (*Epcam*$^{-/-}$), *Epcam*$^{AA/AA}$, *Epcam*$^{Q/Q}$, and *Epcam*$^{L/L}$ mice. **(C)**. Quantification of Alcian blue/PAS-positive goblet cells in the intestines from 5 days old mice. *Epcam*$^{-/-}$and *Epcam*$^{AA/AA}$ mice exhibit villous atrophy in the small intestines and partial loss of goblet cells in the colon, whereas no obvious histological abnormalities were observed in the intestines of *Epcam*$^{Q/Q}$, and *Epcam*$^{L/L}$ mice at this age. Adjusted P values (one-way ANOVA): ** <0.01; *** <0.001; **** <0.0001. Scale bars: (**A, B**) 50 µm.

Development • Supplementary information

## Figure S2

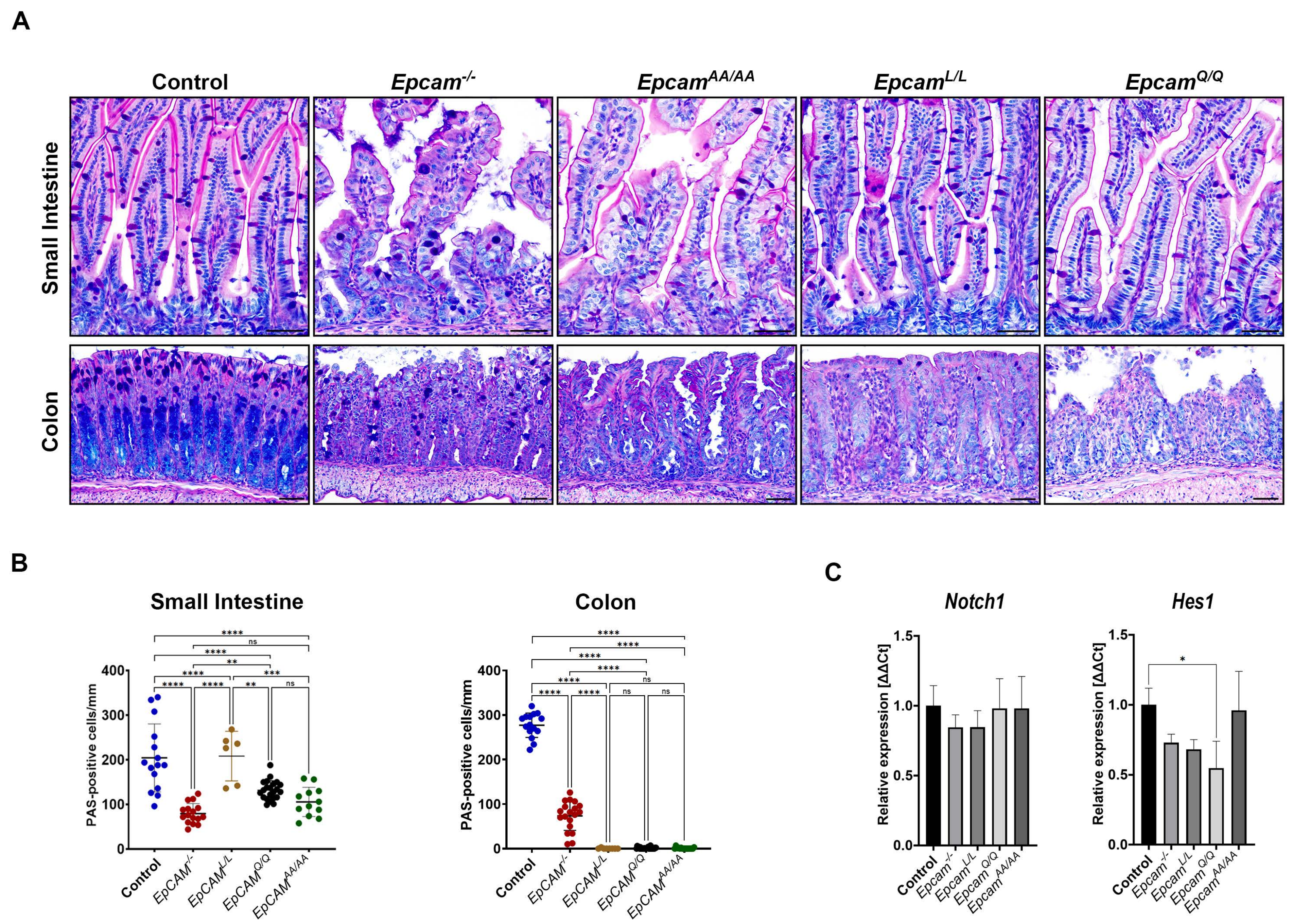


**Fig. S2. Expression of cleavage-resistant EpCAM leads to a decrease in number of mucin-producing goblet cells. (A)**. Representative images (from at least 5 mice per genotype) of Alcian blue/PAS staining of small (top panels) and large (bottom panels) intestines from 21 days old wildtype (Control), EpCAM null (*Epcam*$^{-/-}$), *Epcam*$^{AA/AA}$, *Epcam*$^{Q/Q}$, and *Epcam*$^{L/L}$ mice. **(B)**. Quantification of Alcian blue/PAS-positive goblet cells in the intestines from 21 days old mice. Number of goblet cells was substantially decreased in the small intestines from *Epcam*$^{-/-}$, *Epcam*$^{AA/AA}$, *Epcam*$^{Q/Q}$, but not *Epcam*$^{L/L}$ mice, and virtually eliminated in the large intestines of mice expressing any of the three EpCAM variants. **(C)**. Gene expression of *Notch1* (left) and *Hes1* (right) in the intestines from newborn wildtype (Control), EpCAM null (*Epcam*$^{-/-}$ ), *Epcam*$^{AA/AA}$, *Epcam*$^{Q/Q}$ and *Epcam*$^{L/L}$ mice. Expression of Notch1 was not affected in any of the strains expressing EpCAM variants, whereas expression of Hes1 was slightly decreased in *Epcam*$^{Q/Q}$ *mice. Adjusted P values (one-way ANOVA): * <0.05; ** <0.01; *** <0.001; **** <0.0001. Scale bars: 50 µm.*

Development • Supplementary information

## Figure S3

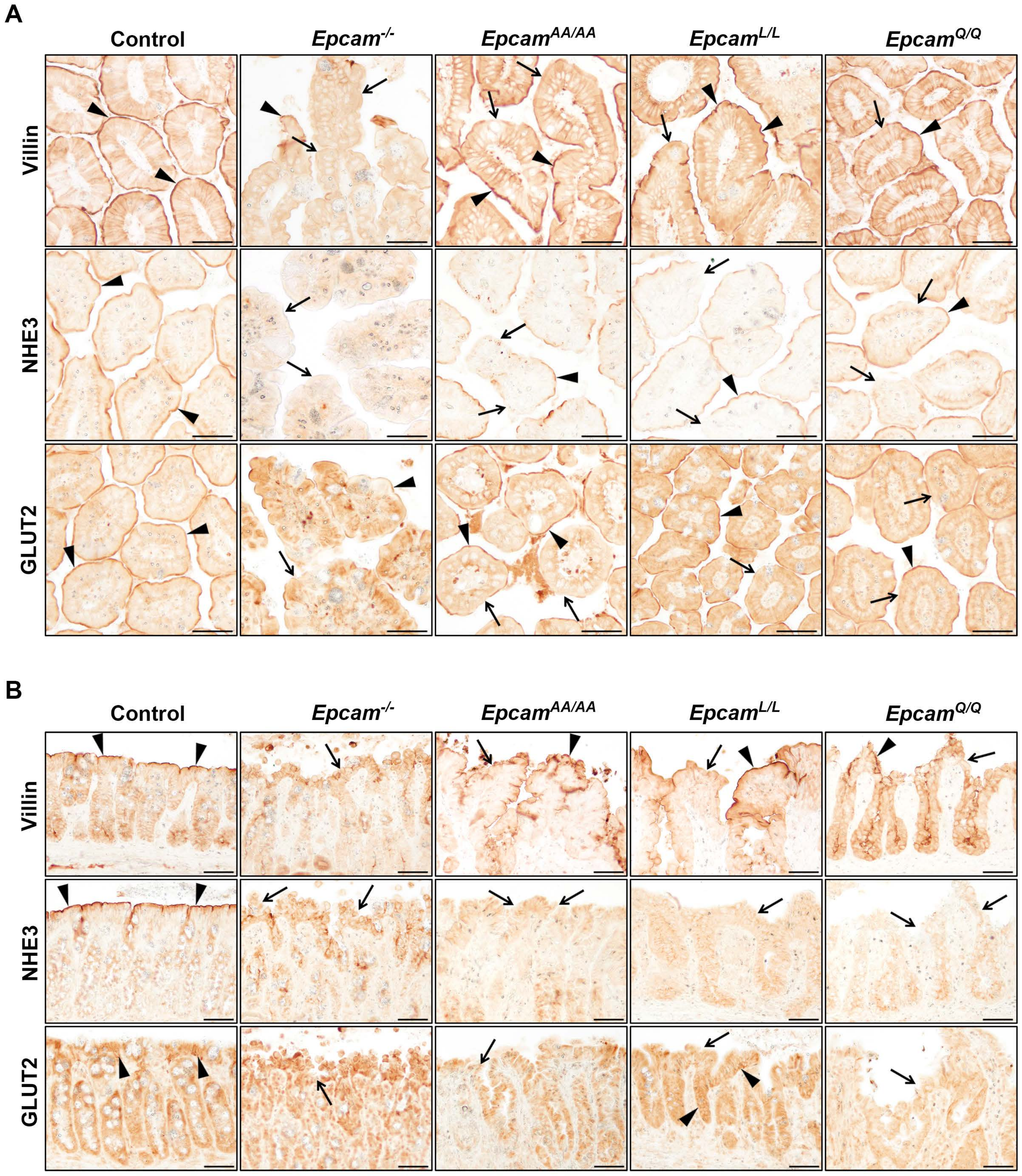


**Fig. S3. Expression of cleavage-resistant EpCAM disrupts membrane localization of intestinal absorption markers. (A, B).** Representative images (from at least 5 mice per genotype) of anti-villin (top), anti-NHE3 (middle), and anti-GLUT2 (bottom) immunostaining of small **(A)** and large **(B)** intestines from 20 days old wildtype (Control), *Epcam*$^{-/-}$, *Epcam*$^{AA/AA}$, *Epcam*$^{L/L}$, and *Epcam*$^{Q/Q}$ mice. Examples of immunostained epithelial surface indicated by arrowheads, lack of staining indicated by arrows. Partial to complete loss of the expression of the three proteins from the intestinal brush border in mice lacking or expressing cleavage-resistant EpCAM. Scale bars: **(A, B)**: 50 µm.

Development • Supplementary information

## Figure S4

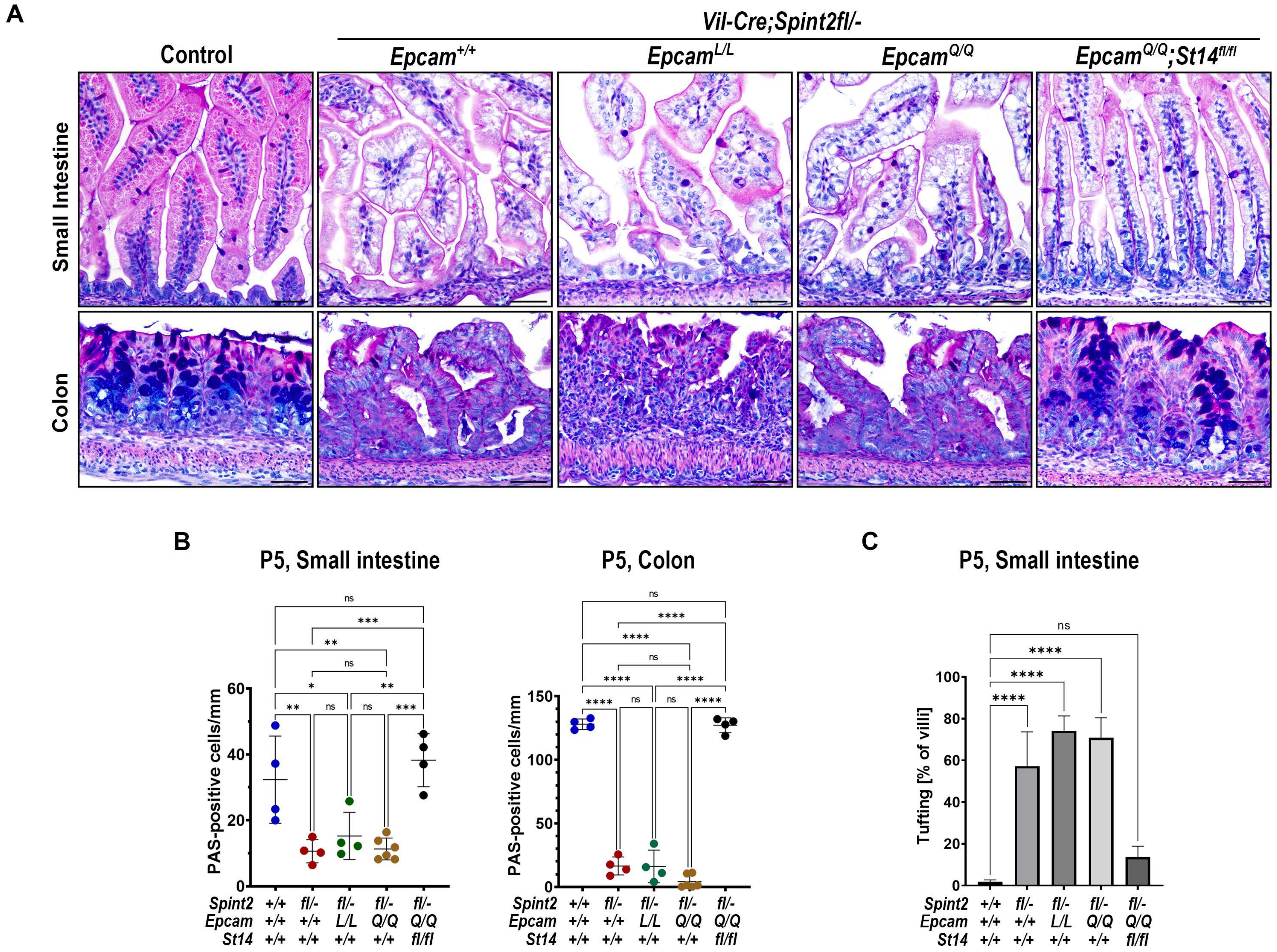


**Fig. S4. Expression of cleavage-resistant EpCAM does not restore number of mucin-producing goblet cells or suppress tufting in HAI-2-deficient intestines.** (A-C). Representative images (from at least 5 mice per genotype) **(A)** and a corresponding quantification **(B)** of Alcian blue/PAS staining of small (top panels) and large (bottom panels) intestines **(A),** and a quantification of tufting (% of villi with visible tufts) in the small intestines **(C)** from 5 days old wildtype (Control), HAI-2-deficient mice expressing wildtype EpCAM (*Vil-Cre*$^{+/0}$*;Spint2*$^{fl/-}$*;Epcam*$^{+/+}$), or homozygous for R80L (*Vil-Cre*$^{+/0}$*;Spint2*$^{fl/-}$;*Epcam*$^{L/L}$), R80Q (*Vil-Cre*$^{+/0}$*;Spint2*$^{fl/-}$;*Epcam*$^{Q/Q}$) EpCAM, and HAI-2/matriptase double-deficient mice homozygous for R80Q EpCAM (*Vil-Cre*$^{+/0}$*;Spint2*$^{fl/-}$*;Epcam*$^{Q/Q}$*;St14*$^{fl/fl}$). Inactivation of matriptase but not the expression of the cleavage-resistant EpCAM, restores formation of goblet cells and suppresses tufting in HAI-2-deficient intestines. Scale bars: 50 µm.

Development • Supplementary information

## Figure S5

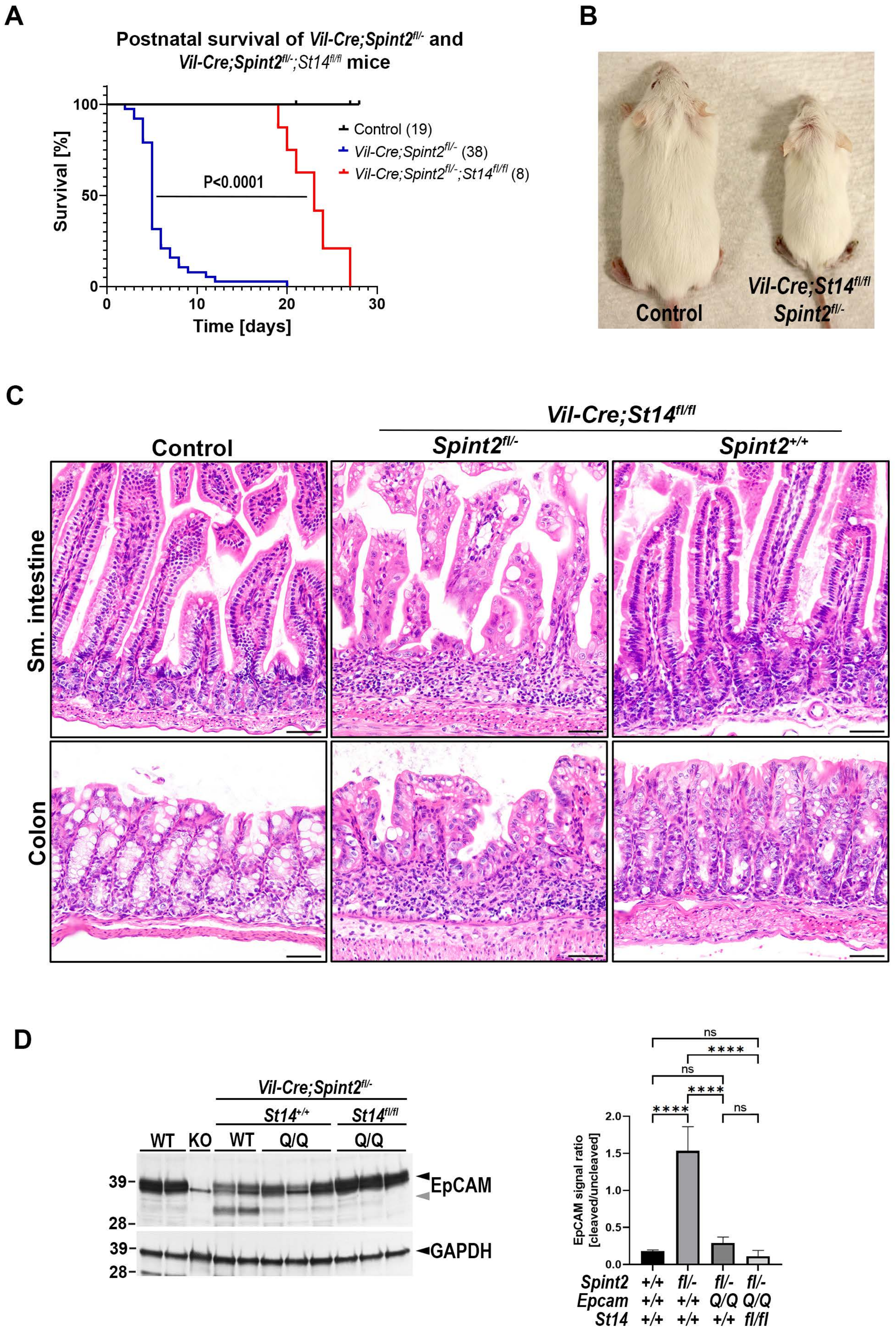


**Fig. S5. Elimination of matriptase delays the onset but does not prevent development of intestinal defects in HAI-2-deficient mice. (A).** Postnatal survival of HAI-2-deficient mice expressing (*Vil-Cre*$^{+/0}$;*Spint2*$^{fl/-}$) or deficient for intestinal matriptase (*Vil-Cre*$^{+/0}$;*Spint2*$^{fl/-}$;*St14*$^{fl/fl}$). Number of mice of each genotype enrolled in the experiment is shown in parenthesis. Elimination of matriptase lead to a substantial increase in overall survival. (B). Representative image (from 4 mice per genotype) of the outward appearance of 21 days old wildtype (Control), and intestinal HAI-2/matriptase double-deficient (*Vil-Cre*$^{/0}$;*Spint2*$^{fl/-}$;*St14*$^{fl/fl}$) mice. Double-deficient mice presented with diarrhea and visibly smaller size. (C). Representative images (from 4 mice per genotype) of H&E staining of small (top panels) and large (bottom panels) intestines from 21 days old wildtype (Control), HAI-2/matriptase double-deficient (*Vil-Cre*$^{+/0}$;*Spint2*$^{fl/-}$;*St14*$^{fl/fl}$) and matriptase single-deficient (*Vil-Cre*$^{+/0}$;*Spint2*$^{+/+}$;*St14*$^{fl/fl}$) mice. Villous atrophy, loss of normal crypt architecture, and stromal inflammation detected in HAI-2-deficient but not HAI-2-expressing mice lacking intestinal matriptase. (D). Representative Western blot (left, out of two independent experiments) and the quantification of the ratio of the signal corresponding to cleaved vs. uncleaved EpCAM (right) in intestines from five days old mice. Inactivation of matriptase appeared to further limit EpCAM cleavage in *Vil-Cre*$^{+/0}$;*Spint2*$^{fl/-}$;*Epcam*$^{Q/Q}$ mice although the difference did not reach statistical significance. Adjusted P values (one-way ANOVA): **** <0.0001. Scale bars: 50 µm.

Development • Supplementary information

**Table S1. Sequences of guide RNAs and genotyping primers used in this study.**

| Gene | Guide target/Primer sequences |
|---|---|
| Gene targeting | |
| *Epcam* | Guide 1: 5'-gaggaggataaagcccgaag-3'<br>Guide 2: 5'-tgactcacagcaagtctggg-3' |
| Screening and genotyping | |
| *Epcam* KO allele | 5'-aatccttggcctggatggc-3'<br>5'-ggaaagctcactcactagg-3' |
| *Epcam* WT allele | 5'-aatccttggcctggatggc-3'<br>5'-cttcgggctttatcctcct-3' |
| *Epcam* R80A/R81A allele | 5'-aatccttggcctggatggc-3'<br>5'-ttcgggctttattgcggcg-3' |
| *Epcam* R80L allele | 5'-aatccttggcctggatggc-3'<br>5'-cttcgggctttatcctaag-3' |
| *Epcam* R80Q allele | 5'-cacagcaagtctgggcag-3'<br>5'-ggaaagctcactcactagg-3' |
| *Spint2* KO allele | 5'-atctgcaacctcaagctagc-3'<br>5'-cagaaccagcaaactgaagg-3' |
| *Spint2* WT allele | 5'-aacacatttcaccaccatgc-3'<br>5'-ccagactttcctaagtggg-3' |
| *Vil-Cre* allele | 5'-ggacatgttcagggatcgccaggcg-3'<br>5'-gcataaccagtgaaacagcattgctg-3' |
| *St14* Flox and WT alleles | 5'-cagtgctgttcagcttcctctt-3'<br>5'-gtggaggtggagttctcatacg-3' |
| Quantitative RT-PCR | |
| *Notch1* | 5'-gcaactgtccttctgccatatac-3'<br>5'-gtcttcagactccttgcatacc-3' |
| *Hes1* | 5'-caacacgacaccggacaaac-3'<br>5'-cggaggtgcttcacagtcat-3' |
| *GAPDH* | 5'-caaggtcatccatgacaactttg-3'<br>5'-gtccaccaccctgttgctgtag-3' |

**Table S2. Sequences of CRISPR donor ssDNA oligos used in this study.**

| Intended mutation | Oligo ssDNA sequences |
|---|---|
| *Epcam* R80A/R81A | tgaagccgcggctgtctctcattgggcgttactgtcatcgcctttcca gtggcgtctaaatgcttggcgatgaaagcagaaatgactcacagca agtctggcgccgcaataaagcccgaaggggcgatccagaacaac gatgggctgtacgaccccgactgcgacgagcaggggctcttcaaa gccaagcagtgcaacgg |
| *Epcam* R80L | tgaagccgcggctgtctctcattgggcgttactgtcatcgcctttcca gtggcgtctaaatgcttggcgatgaaagcagaaatgactcacagca agtctgggcttaggataaagcccgaaggggcgatccagaacaac gatgggctgtacgaccccgactgcgacgagcaggggctcttcaaa gccaagcagtgcaacgg |
| *Epcam* R80Q | tgaagccgcggctgtctctcattgggcgttactgtcatcgcctttcca gtggcgtctaaatgcttggcgatgaaagcagaaatgactcacagca agtctgggcagaggataaagcccgaaggggcgatccagaacaac gatgggctgtacgaccccgactgcgacgagcaggggctcttcaaa gccaagcagtgcaacgg |

**Table S3. List of antibodies used in the study**

| | | | |
|---|---|---|---|
| **Immunohistochemistry** | | | |
| **Primary** | | | |
| **Antigen** | **Manufacturer** | **Cat. No.** | **Concentration** |
| EpCAM | R&D Systems, Minneapolis, MN | AF960 | 5 ug/ml |
| Claudin-7 | Life Technologies, Rockford, IL | 34-9100 | 2.5 ug/ml |
| Villin | Santa Cruz Biotechnology, Santa Cruz, CA | Sc-7672 | 2.0 ug/ml |
| NHE3 | Novus Biologicals, Centennial, CO | NBP1-82574 | 1.0 ug/ml |
| GLUT2 | Novus Biologicals, Centennial, CO | NBP1-87581 | 1.0 ug/ml |
| **Secondary** | | | |
| Anti-rabbit | Vector Laboratories, Burlingame, CA | BA-1000 | 2.5 ug/ml |
| Anti-goat | Vector Laboratories, Burlingame, CA | BA-9500 | 2.5 ug/ml |
| **Western Blot/Immunoprecipitation** | | | |
| **Primary** | | | |
| HAI-2 | R&D Systems, Minneapolis, MN | AF1106 | 1 ug/ml |
| EpCAM | R&D Systems, Minneapolis, MN | AF960 | 1 ug/ml |
| Claudin-7 | Life Technologies, Rockford, IL | 34-9100 | 0.5 ug/ml |
| HA | Cell Signaling Technology, Danvers, | 3724 | 0.5 ug/ml |
| Myc | MA Cell Signaling Technology, | 2276 | 0.5 ug/ml |
| GAPDH | Danvers, MA Abcam, Cambridge, MA | ab9485 | 0.5 ug/ml |
| **Secondary** | | | |
| Anti-mouse | Dako Cytomation, Carpinteria, CA | D0486 | 1 ug/ml |
| Anti-rabbit | Dako Cytomation, Carpinteria, CA | D0487 | 1 ug/ml |
| Anti-goat | Sigma, St.Louis, MO | A4187 | 1 ug/ml |

Development • Supplementary information
